# A predictive model for cognitive decline using social determinants of health

**DOI:** 10.1016/j.jarlif.2025.100056

**Published:** 2026-01-06

**Authors:** Yingnan He, Yu Leng, Ana-Maria Vranceanu, Christine S. Ritchie, Deborah Blacker, Sudeshna Das

**Affiliations:** a Department of Neurology, Massachusetts General Hospital, Boston, MA, 02114, USA; b Harvard Medical School, Boston, MA, 02115, USA; c Department of Psychiatry, Massachusetts General Hospital, Boston, MA, 02114, USA; d Mongan Institute Center for Optimal Aging and Serious Illness Research and the Division of Palliative Care, Aging, and Geriatric Medicine, Massachusetts General Hospital, Boston, MA, 02114, USA; e Department of Epidemiology, Harvard T.H. Chan School of Public Health, Boston, MA, 02115, USA

**Keywords:** Mexican health and aging study (MHAS), Cognitive decline, Aging, Social determinants of health (SDoH), Health disparities, predictive modeling, Machine learning, Stacked model, Bias analysis, Interpretable models

## Abstract

**Background:**

Early diagnosis of Alzheimer’s disease and related dementias (AD/ADRD) is critical but often constrained by limited access to fluid and imaging biomarkers, particularly in low-resource settings.

**Objective:**

To develop and evaluate a predictive model for cognitive decline using survey-based data, with attention to model interpretability and fairness.

**Methods:**

Using data from the Mexican Health and Aging Study (MHAS), a nationally representative longitudinal survey of adults aged 50 and older (*N* = 4095), we developed a machine learning model to predict future cognitive scores. The model was trained on survey data from 2003 to 2012, encompassing demographic, lifestyle, and social determinants of health (SDoH) variables. A stacked ensemble approach combined five base models—Random Forest, LightGBM, XGBoost, Lasso, and K-Nearest Neighbors—with a Ridge regression meta-model.

**Results:**

The model achieved a root-mean-square error (RMSE) of 39.25 (95 % CI: 38.12–40.52), representing 10.2 % of the cognitive score range, on a 20 % held-out test set. Features influencing predictions, included education level, age, reading behavior, floor material, mother’s education level, social activity frequency, the interaction between the number of living children and age, and overall engagement in activities. Fairness analyses revealed model biases in underrepresented subgroups within the dataset, such as individuals with 7–9 years of education.

**Discussion:**

These findings highlight the potential of using accessible, low-cost SDoH survey data for predicting risk of cognitive decline in aging populations. They also underscore the importance of incorporating fairness metrics into predictive modeling pipelines to ensure equitable performance across diverse groups.

## 1 Introduction

Cognitive decline is a critical public health concern affecting aging populations worldwide. Conditions such as Alzheimer’s disease and related dementias (AD/ADRD) pose significant burdens on individuals, families, and healthcare systems [1]. Early detection of dementia allows for timely interventions and care strategies that can alleviate symptoms and enhance the well-being of both those affected and their families [2,3]. However, identifying who should undergo further cognitive evaluation remains challenging. Simple screening tools are often underutilized, and more definitive diagnostic methods, such as neuroimaging or fluid biomarkers [4], are expensive and typically reserved for individuals already showing clinical symptoms. Access to these biomarkers is even more limited in low-resource settings due to structural barriers and socioeconomic disparities.

Given these limitations in current diagnostic approaches, researchers have increasingly turned to machine learning methods to improve early detection of cognitive decline [[5], [6], [7]]. However, most of these models rely heavily on healthcare records or biomarker data. In parallel, emerging evidence has highlighted the importance of health (SDoH) factors—such as education, economic conditions, and social interaction—in influencing late-life cognitive outcomes [8,9]. For example, lower education is associated with cognition in later life [10]. Social engagement is another important factor that plays a protective role in cognitive health, and frequent social interactions have been associated with a slower rate of cognitive decline [11]. The Mexican Health and Aging Study (MHAS) [12] and Mex-Cog [13], which collect data on health, demographics, socioeconomic status, disability/functionality/frailty, social/family support and cognition, provides a valuable resource for examining the impact of SDoH on cognition [[14], [15], [16], [17], [18], [19], [20], [21], [22], [23], [24], [25]]. For example, studies using MHAS demonstrated that educational attainment predicts cognitive performance [22]; spousal and offspring education are associated with cognition [21,24]; and cognitive benefits of education may vary by gender and cohort over time [23]. Others have demonstrated social interaction interdependence on cognition among couples [20], and that social isolation—such as having an adult child migrate to the U.S.—was linked to steeper memory decline among older adults, especially women [14].

In this study, we developed a model to predict cognitive function using SDoH data from MHAS [12]. Our approach uses a stacking ensemble of machine learning models trained exclusively on survey-based data (encompassing demographics, economic status, health history, lifestyle, and social interaction) collected in 2003 and 2012 to predict cognition in 2016 and 2021. Additionally, we analyzed model interpretability and fairness, leveraging SHapley Additive exPlanations (SHAP)[26] values for explainability and conducting subgroup bias analyses to assess predictive equity.

## 2 Methods

### 2.1 Dataset

This study used a subset of the MHAS [12] dataset released by the DrivenData [27] PREPARE Challenge (Pioneering Research for Early Prediction of Alzheimer's and Related Dementias EUREKA Challenge) Social Determinants Track. The MHAS data were originally collected through in-person interviews conducted by trained interviewers across multiple waves. In this study, we were provided with data from the 2003 and 2012 waves. It covered a wide range of topics related to health, aging, and social and economic circumstances, including income, employment, and lifestyle factors (see Supplementary Table 1 for full list of variables). These variables served as the raw input features in our model.

The outcome variable was a composite cognitive function score assessed in 2016 and 2021 as part of the MHAS Cognitive Aging Ancillary Study (Mex-Cog) [13]. This score, ranging up to a maximum of 384, was derived from detailed in-person cognitive assessments, with higher values indicating better cognitive performance. The goal of the model was to predict future cognitive performance 4 and 9 years into the future, based on social, demographic, and health-related data collected in 2003 and 2012.

### 2.2 Data preprocessing and feature engineering

To prepare the dataset for modeling, we conducted data cleaning, feature encoding, and the creation of additional variables that capture individual changes and summary metrics. This step aimed to enhance data quality and represent domain-relevant features for machine learning models.

We first cleaned and processed the data to ensure it was suitable for modeling. Nine variables were removed due to having >70 % missing data. These included employment-related variables (e.g., job end year and reason), and U.S. migration variables (e.g., year of first migration, total years in the U.S., job type in the U.S., and residency status), primarily from the 2012 interview. For the remaining missing data, we used mean, median, or mode imputation based on the feature type. Categorical variables were encoded into numeric form. Binary variables (e.g., sex) were encoded as 0 or 1, and ordinal variables (e.g., flooring type: Wood/Mosaic, Concrete, Mud) were assigned increasing integers to reflect relative costs. We also created indicators for age in 2012 (e.g., over 60, 70, or 80). Additionally, we calculated differences between 2003 and 2012 features to reflect individual changes over time. These differences were computed only for numeric and ordinal variables. To identify significant interaction terms, we tested all possible feature pairs using Ordinary Least Squares (OLS) regression and selected significant interaction terms based on the following criteria: p-value < 0.01, absolute β coefficient > 0.5, and R-squared value > 0.2.

Additionally, we created summary features. 1) ADL: We summed difficulties (e.g., eating, bathing) and added binary indicators for any difficulty. 2) Emotional well-being: We counted positive (e.g., happy, energetic) and negative emotions (sad, tired, etc.), then calculated emotional scores and binary flags for each year. 3) Activity engagement: We computed a total activity score in 2012 from participation in reading, sewing, games, TV watching, housework, and communication. Details of engineered summary features are listed in Supplementary Table 2. The year of cognitive assessment (2016 or 2021) was also included as a feature in both the base models and the meta-model to account for time-related changes in cognitive outcomes. Cognitive scores from both 2016 and 2021 were modeled jointly in a single pooled dataset, with the year variable allowing the model to distinguish between the two assessment waves. The dataset was split into 80 % for training and 20 % for the held-out test set. This comprehensive feature engineering ensured that both static and dynamic aspects of aging were captured prior to training the prediction models.

### 2.3 Machine learning and statistical analysis

**Fig. 1:**
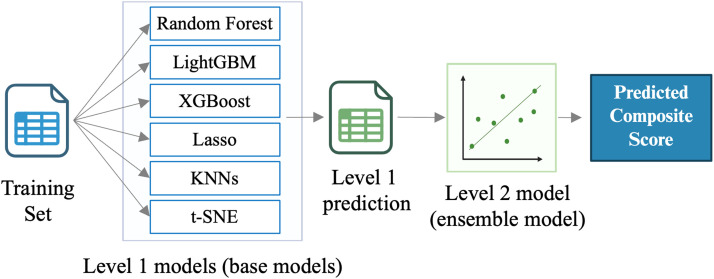
Architecture of the Stacking Ensemble Model. The first layer consists of a collection of base models, which capture both linear and non-linear patterns in the training data.

After data preprocessing, a two-layer stacking ensemble modeling approach was employed (Fig. 1). The first layer consisted of LightGBM [28], XGBoost [29], Random Forest, Lasso regression, and multiple K-Nearest Neighbors (KNN) models trained on different feature groupings and two t-SNE embeddings.

• LightGBM and XGBoost are gradient boosting decision tree models well-suited for handling high-dimensional, tabular data with complex interactions.
• Random Forest is a bagging-based ensemble method that provides robust predictions and helps reduce overfitting.
• Lasso regression, a linear model with L1 regularization, was included for its ability to perform automatic feature selection and capture interpretable linear relationships.
• K-Nearest Neighbors (KNN) is a non-parametric algorithm that captures localized, non-linear patterns in the data.
• t-SNE a nonlinear dimensionality-reduction method for high-dimensional data was to capture latent variables in the data.

This integration of both linear and non-linear models handles mixed variable types and is robust to overfitting. LightGBM and XGBoost were trained using a 5-fold cross-validation approach, with hyperparameters optimized through the Hyperopt library [30]. To improve the model’s generalizability and reduce potential biases towards training set, instance weights were used during training LightGBM and XGBoost. These weights were derived by training a logistic regression classifier to distinguish between the training and test datasets based on variables and then using the predicted probabilities as instance weights. For Random Forest, hyperparameter tuning was performed using grid search with 5-fold cross-validation to find a best combination of hyperparameters. For Lasso regression, we used grid search to find best regularization strength (alpha) with 5-fold cross-validation. We used KNNs configured with various values of K (*K* = 2, 4, 8, 16, 32, 64) for all features to explore non-linear patterns in the data. Additionally, we grouped features into nine non-overlapping categories—demographic, health status, mental health, diagnosis history, lifestyle, wealth, healthcare use, decision making, and life changing—and generated separate KNN outputs for each category, see category details in Supplementary Table 1. Based on the cross-validation results during training, we determined that four categories—demographic, decision making, wealth, and lifestyle variables—provided improvements to the second-layer ensemble model’s performance. Two components derived from t-Distributed Stochastic Neighbor Embedding (t-SNE) [31] embeddings were utilized as features to capture latent patterns in the high-dimensional feature space. We also explored other dimensionality reduction techniques, such as Principal Components Analysis (PCA) and Uniform Manifold Approximation and Projection (UMAP) [32]. However, these techniques did not yield improvements in model performance and were not included in the final approach.

The predictions from the Level 1 models were combined into a new training set, which serves as input for the second-level model. The Level 2 model further refines predictions by combining outputs from the base models, ultimately generating the final composite score predictions. The second layer was a Ridge regression model (a regularized linear regression method that shrinks coefficients to prevent overfitting) integrating predictions from the base models, two t-SNE embeddings, and the year of composite score (2016 or 2021). We performed a grid search with five-fold cross-validation to determine the optimal alpha for the Ridge model, testing values over a logarithmic range (10⁻⁴ to 10²). Specifically, the training set was resampled with replacement to create 50 bootstrap samples, and a Ridge model was trained on each sample. For each test data point, predictions were generated from all 50 models and then averaged to produce the final prediction, ensuring greater robustness and reducing variability in the results. We also experimented with using a LightGBM regressor as the meta-model with hyperparameter tuning. Despite achieving very low RMSE during training, the model performed poorly on the held-out test set, suggesting that the LightGBM meta-model was prone to overfitting. This reinforced our choice of Ridge regression as a more stable and generalizable meta-learner.

For all models requiring hyperparameter tuning, LightGBM, XGBoost, Random Forest, Lasso regression in the first layer, and Ridge regression as the ensemble model, root-mean-square deviation (RMSE) was used as the metric to select the best hyperparameters, ensuring consistent evaluation and optimal performance across different models. The full search space and the selected hyperparameters for each model are summarized in Supplementary Table 3. To estimate uncertainty, we computed the 95 % confidence interval using 1000 bootstrap resamples from the held-out test set.

Robustness analyses were conducted by permuting top ten features with the highest SHAP values of LightGBM model to test the model’s stability and identify its reliance on specific inputs. The impact of these changes on model performance, measured by RMSE on held-out test data, provided insights into feature importance and resilience to minor variations in data.

### 2.4 Error and bias analysis

We conducted error analysis by examining a scatter plot of residuals (true values minus predicted values) against the predicted composite score to assess whether the prediction errors were randomly distributed. We visualized the distributions of residuals for years of 2016 and 2021 to compare model performance and conducted a *t*-test on the predicted scores between the two years to assess whether prediction accuracy differed significantly across timepoints. We then used an OLS regression model to examine the relationship between residuals and the top six features selected from the top SHAP-ranked features of LightGBM model. The model was fitted with these features as independent variables and the residuals as the dependent variable. This analysis aimed to assess whether a specific feature systematically influenced the magnitude or direction of the model’s prediction errors.

For bias analysis, we first compared the predicted composite scores against the true scores across subgroups defined by six key features. By plotting score distributions and conducting paired *t*-tests, we identified subgroups with significant differences between predicted and actual scores.

## 3 Results

In this section we report descriptive characteristics of the study population; model performance and interpretability; as well as model error and bias analysis. Model evaluation focused on RMSE on a held-out test set, feature importance, systematic errors, and subgroup-level biases. These results demonstrate the effectiveness and fairness of our modeling approach across a range of subpopulations.

### 3.1 Participants

The final analytic sample (*n* = 4095) was derived from MHAS participants who completed surveys in both 2003 and 2012 and had valid cognitive scores in 2016 or 2021. The characteristics of the dataset are summarized in Table 1. Females accounted for 57.7 % (*n* = 2364) of the dataset, and individuals aged 60–69 years comprised the largest age group, representing 42.3 % (*n* = 1734). The majority of participants were married or in a civil union, making up 65.1 % (*n* = 2264) of the total sample. Regarding educational level, individuals with six or fewer years of schooling formed the majority, accounting for 70.2 % (*n* = 2875) of the dataset.

**Table 1:** Demographic and Socioeconomic Characteristics of the MHAS Dataset.

Characteristics	Total	Train set	Test set
**Sex, n ( %)**			
Male	1731 (42.3)	1401 (42.8)	330 (40.3)
Female	2364 (57.7)	1875 (57.2)	489 (59.7)
**Age group, n ( %)**			
<= 49	6 (0.15)	6 (0.2)	0 (0.0)
50 - 59	1163 (28.4)	946 (28.9)	217 (26.5)
60 - 69	1734 (42.3)	1374 (41.9)	360 (44.0)
70 - 79	857 (20.9)	686 (20.9)	171 (20.9)
>= 80	219 (5.3)	174 (5.3)	45 (5.5)
Unknown	116 (2.8)	90 (2.7)	26 (3.2)
**Marriage Status, n ( %)**			
Married	2664 (65.1)	2137 (65.2)	527 (64.3)
Divorced	344 (8.4)	280 (8.5)	64 (7.8)
Widowed	779 (19.0)	614 (18.7)	165 (20.1)
Single	194 (4.7)	156 (4.8)	38 (4.6)
Unknown	114 (2.8)	89 (2.7)	25 (3.1)
**Educational level, n ( %)**			
None	726 (17.7)	556 (17.0)	170 (20.8)
1–5 years	1327 (32.4)	1111 (33.9)	216 (26.4)
6 years	822 (20.1)	636 (19.4)	186 (22.7)
7–9 years	658 (16.1)	515 (15.7)	143 (17.5)
10+ years	436 (10.6)	358 (10.9)	78 (9.5)
Unknown	126 (3.1)	100 (3.1)	26 (3.2)

### 3.2 Model performance and feature importance

**Fig. 2:**
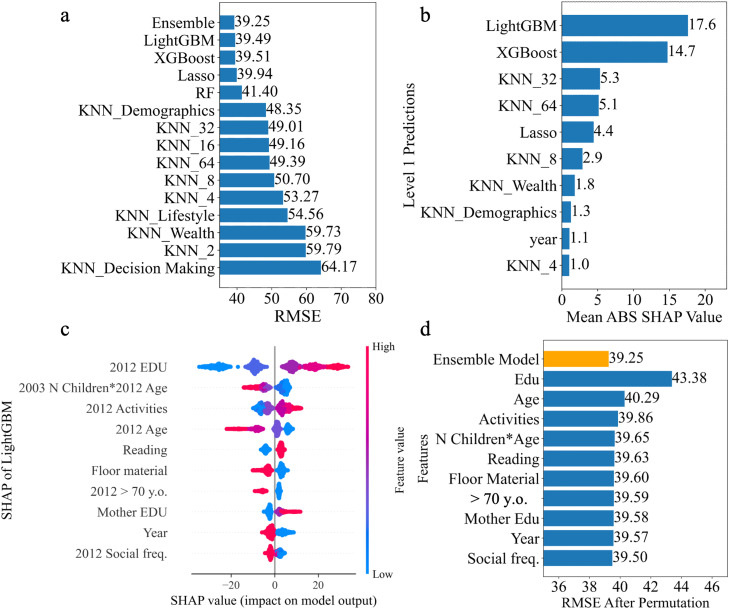
Model Performance Analysis. (a) Comparison of RMSE for the ensemble model and individual base models, the ensemble model achieves the lowest RMSE. (b) Mean absolute SHAP values of Level 1 model predictions, LightGBM and XGBoost exhibit the highest SHAP values. (c) Top 10 features influencing the LightGBM model predictions as shown by SHAP summary plot. Features are ranked by importance, with education level being the most influential. (d) Impact of feature permutation on model performance as measured by RMSE. Each subsequent bar shows the RMSE after permuting one of the top features identified through SHAP analysis. Permuting education level caused the largest increase in RMSE, followed by feature of age. RF = Random Forrest; y.o. = years old; Social freq. = Social frequency; EDU = Education.

To predict future cognition, we tested the various models on the on the held-out test set (*n* = 819), representing 20 % of the full dataset. The ensemble model (see Fig. 1) achieved the best performance, with RMSE of 39.25 (95 % CI: 38.12–40.52), compared to individual base models (Fig. 2a). This level of error (39 points) represents approximately 10 % on the 384-point scale, indicating that the model can capture broad cognitive trends. Each base model was evaluated separately, with RMSEs ranging from 39.49 (LightGBM) to 64.17 (KNN of decision-making features). While the ensemble model’s improvement over LightGBM was modest, the stacked approach enhanced robustness through model averaging and helped mitigate overfitting, as evidenced by stability in permutation-based sensitivity analyses. LightGBM alone may be preferable for simpler, resource-constrained settings, but the ensemble framework offers greater resilience and flexibility for future extensions. We also evaluated the contribution of each base model to the ensemble model using SHAP analysis. The top 10 base models influencing the ensemble predictions are shown in Fig. 2b The LightGBM displayed the highest absolute SHAP values, which aligns with its individual performance achieving the lowest RMSE among the base models.

Using SHAP analysis on the LightGBM model, we identified six features that had the highest influence on predictions (Fig. 2c), including education level, age, reading behavior, floor material (as a proxy for economic status), mother’s education level, and social activity frequency. Many of these variables align with established SDoH domains. For example, education level and mother’s education reflect Education Access and Quality; floor material serves as a proxy for Economic Stability; and social activity frequency map to Social and Community Context, as defined by the SDoH constructs in Healthy People 2030[33]. Among the top-ranked engineered features were the interaction between the number of living children and age, an overall activity engagement, and whether individuals were over 70 years old in 2012. The year of cognitive assessment (2016 or 2021) is also one of the top 10 features of LightGBM model.

Next, the robustness analysis revealed that permuting the features of education level and age caused a noticeable increase in RMSE (Fig. 2d). This indicates that our model relies heavily on these features to make accurate predictions. The reliance on these features underscores their importance in predicting the composite cognition score but also highlights the potential risk of overfitting if these features are not well-represented or balanced across subpopulations in the dataset.

### 3.3 Error analysis

Next, we examined if there were systematic errors in the model by analyzing the distribution of residuals against predicted values, comparing results across two cognitive testing years, and evaluating correlations with key features.

**Fig. 3:**
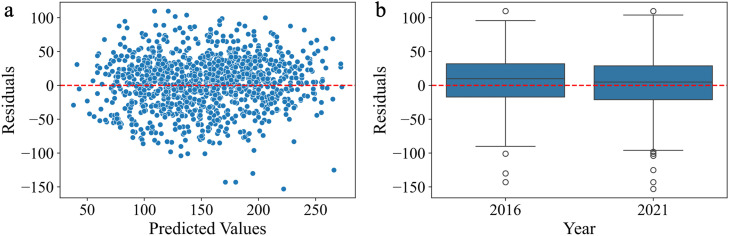
Model Evaluation: Residual Patterns. (a) Residuals vs. predicted values: the residuals are randomly scattered around zero, indicating that the model does not exhibit systematic bias across the range of predicted values. (b) Residuals by year (2016 vs. 2021): there is increased variability in model predictions for 2021. The model tends to underestimate true composite scores more often for 2016.

Since the residuals appear randomly distributed around the horizontal axis (Fig. 3a), the model does not exhibit systematic error trends in its prediction. The spread of residuals is slightly larger for 2021 compared to 2016 (Fig. 3b), suggesting that the model error tends to increase with extension of the prediction timeline. The median residual for 2016 is slightly higher than that for 2021, indicating greater mean prediction error in 2016. This may be attributable to the larger number of training data points available for 2021—approximately twice as many as for 2016. A *t*-test comparing predicted scores between the two years showed a statistically significant difference (*t* = 2.207, *p* = 0.03), suggesting that year-related differences in data availability or distribution may have influenced model predictions.

To assess whether systematic errors were associated with specific features, we selected six SDoH variables from the top six SDoH SHAP-ranked features of the LightGBM model, then conducted a regression using these features as independent variables and the residuals as the dependent variable. The results revealed that these features explained only a minimal proportion of the variance in residuals (R² = 0.008), indicating that the residuals were not strongly related to these variables. None of the features demonstrated a statistically significant relationship with the residuals, suggesting that the model’s errors are not strongly biased toward any of these SDoH features. However, a few features had relatively larger coefficients—for instance, individuals who reported a reading habit (coefficient = 2.10, *p* = 0.427) or had higher maternal education levels (coefficient = 1.27, *p* = 0.473) tended to have slightly higher residuals, though these associations were not statistically significant.

### 3.4 Bias analysis

**Fig. 4:**
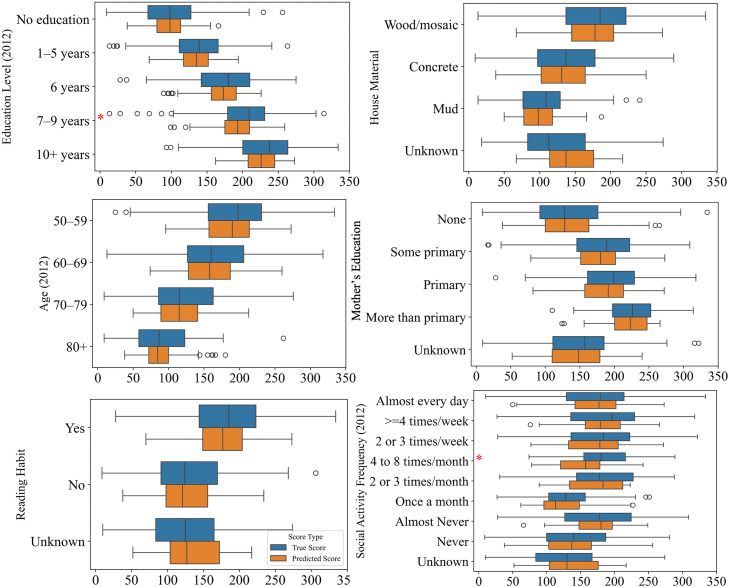
Model Evaluation: Bias Analysis. The model significantly underpredicts composite scores for two underrepresented subgroups: individuals with 7–9 years of education and those who engage in social activities 4–8 times per month.

Next, we examined potential prediction bias across various subgroups by plotting the distributions of true and predicted cognitive scores and performing paired *t*-tests for the top six model features (Fig. 4). The results revealed significant discrepancies in two subgroups: the model underpredicted scores for individuals with 7–9 years of education and social activity level of 4–8 times per month.

Further, we examined the distribution of the subgroups within the dataset. Individuals with 7–9 years of education represented 16.1 % (*n* = 658). Individuals with fewer than six years of education made up 70.2 % (*n* = 2875) of the sample, indicating that the model was trained primarily on individuals with education levels lower than 7 years. This imbalance likely contributed to the model’s tendency to underpredict cognitive scores in individuals with higher education. Similarly, social activity levels were concentrated at the extremes: 22.6 % (*n* = 926) of participants reported engaging in social activities almost every day, while 45.4 % (*n* = 1861) reported never doing so. In contrast, middle-frequency subgroups were rare—only 5.0 % (*n* = 207) reported engaging 4–8 times per month. These findings suggest that model biases likely stem from the underrepresentation of the two subgroups in the modeling dataset: individuals with 7–9 years of education and those engaging in social activities 4–8 times per month. This underrepresentation may result from overly granular survey categories or limited recruitment, both of which could reduce generalizability and contribute to biased performance.

## 4 Discussion

In this study, we developed an ensemble machine learning model to predict cognitive scores using demographics, health history, lifestyle behaviors, and social determinants of health variables from the MHAS cohort. The model achieved an RMSE of 39.25 on a held-out test set, indicating strong performance in capturing population-level cognitive trends. Through SHAP-based interpretability, we identified six features influencing predictions, including education level, age, reading behavior, floor material, mother’s education level, and social activity frequency, as well as three engineered features: the interaction between the number of living children and age, an overall activity engagement, and whether individuals were over 70 years old in 2012. These findings offer practical public health insights. The identified predictors may inform risk stratification and support low-cost, community-level interventions. For instance, individuals with low educational attainment or limited engagement in daily activities—both associated with lower predicted cognitive scores—could be prioritized for early screening or cognitive stimulation programs. Moreover, economic indicators, such as floor material, may help policymakers identify underserved communities with structural disadvantages, including healthcare access.

This work contributes to growing efforts to tailor health research to populations historically underrepresented in studies of cognitive decline. Our findings align with several recent studies that applied machine learning to predict cognitive outcomes using SDoH. Kindo et al. used MHAS and Mex-Cog data to forecast cognitive decline with tree-based models, also identifying education and social engagement as influential predictors [34]. Poudel et al. demonstrated that sociodemographic factors alone could effectively predict cognitive status in an Australian cohort [35], and Singh et al. found that psychosocial and economic factors were more predictive of cognitive function than physical activity or environmental measures in Canadian older adults [36]. Building on this work, our stacking ensemble framework offers a generalizable and interpretable model tailored to the Mexican aging population. Notably, it extends prior approaches by predicting continuous cognitive scores for greater granularity and by incorporating fairness-aware analyses to evaluate equity across subgroups.

Recent studies have raised concerns about algorithmic bias in predictive models, where underrepresented subgroups may receive systematically inaccurate predictions due to imbalanced training data or overlooked contextual factors [[37], [38], [39]]. Our model evaluation revealed prediction biases in two subgroups: individuals with 7–9 years of education and social activity levels of 4–8 times per month. Both were systematically underpredicted (predicted with lower cognitive score than actual). To mitigate these disparities, we explored three data augmentation strategies: (1) replicating existing data with slight perturbations, (2) duplicating samples from underrepresented subgroups two or three times, and (3) increasing the instance weights of individuals in underrepresented subgroups during training. Despite these mitigation efforts, disparities in prediction accuracy remained for the affected subgroups, highlighting issues with subgroup representation within the training data. Future research may benefit from implementing more targeted methodological strategies (e.g., subgroup-specific calibration, adversarial debiasing, or transfer learning) to enhance fairness and predictive equity across educational and social engagement strata.

Our study has several limitations. First, since the model learns from patterns in the training dataset, it may reinforce dataset imbalances, leading to systematic biases for underrepresented subgroups. To address this, future implementations should include uncertainty measures, such as confidence intervals, to signal when predictions are less reliable, especially for these subgroups. Second, the MHAS waves design introduced some limitations. The lack of data collection between 2003 and 2012 reduced temporal resolution, while the 2012 replenishment sample (absent in 2003) may have introduced cohort effects. Third, including the cognitive assessment year (2016 or 2021) as a predictor constrains the model’s ability to predict risk over different time periods. Future work could develop separate models for short- and long-term risk predictions. Lastly, our selection of SDoH variables was guided by data availability in MHAS rather than formal frameworks such as Healthy People 2030[33]. Future work could enhance conceptual rigor by mapping predictors to established SDoH domains. Predictive performance may also be improved by incorporating additional modalities, such as physical function, healthcare utilization, and/or biomarker data.

Overall, this work demonstrates that demographics, lifestyle, and social determinants of health data, combined with machine learning, can help identify individuals at risk of cognitive decline—offering a scalable and efficient alternative to traditional screening. These data are easy to collect through surveys or phone interviews and provide a cost-effective, participant-friendly option, especially in settings with limited access to specialized clinics or advanced biomarker testing.

## Fundings

We acknowledge funding from NIA P30 AG062421 (Das, Ritchie, Blacker, Vranceanu). This research uses data from the Mexican Health and Aging Study (MHAS), which is partly sponsored by the National Institutes of Health/National Institute on Aging (grant number NIH R01AG018016) in the United States and the Instituto Nacional de Estadística y Geografía (INEGI) in Mexico. The authors are grateful to the MHAS research team and participants.

## Data statement

This research uses de-identified secondary data from the Mexican Health and Aging Study (MHAS), which is publicly available. As such, participant consent was obtained during the original data collection, and additional consent was not required for this research.

## Declaration of the use of generative AI and AI-assisted technologies

During the preparation of this work, the authors used OpenAI’s ChatGPT to assist with language. After using this tool, the authors reviewed and edited the content as needed and take full responsibility for the content of the publication.

## CRediT authorship contribution statement

**Yingnan He:** Writing – original draft, Visualization, Methodology, Formal analysis, Data curation. **Yu Leng:** Writing – review & editing, Visualization. **Ana-Maria Vranceanu:** Writing – review & editing. **Christine S. Ritchie:** Writing – review & editing. **Deborah Blacker:** Writing – review & editing. **Sudeshna Das:** Writing – review & editing, Supervision, Resources, Funding acquisition, Conceptualization.

## Declaration of competing interest

The authors declare that they have no known competing financial interests or personal relationships that could have appeared to influence the work reported in this paper.

## Appendix Supplementary materials

Image, application 1
