# Supplementary material for: A predictive model for cognitive decline using social determinants of health
Source: JAR Life. 2026 Jan 6;15:100056. doi: 10.1016/j.jarlif.2025.100056 (PMC12809124; doi:10.1016/j.jarlif.2025.100056)
Supplement: Supplementary file 1 [file mmc1.docx]

# Supplementary Tables

| **Categories** | **Feature** |
| --- | --- |
| Demographics | Binned age group (2003 and 2012) |
|  | Locality size (urban vs. rural) (2003 and 2012) |
|  | Marital status (2003 and 2012) |
|  | Number of marriages (2003 and 2012) |
|  | Binned education level (2012) |
|  | Binned number of living children (2003 and 2012) |
|  | Has lived or worked in the U.S. (2003 and 2012) |
|  | Gender (2003; 2012) |
|  | Mother’s education level (2012) |
|  | Father’s education level (2012) |
|  | Spouse’s gender (2003 and 2012) |
|  | Speaks English (2012) |
|  | Floor material of residence (2012) |
|  | Binary indicator for being above age 60, 70, or 80 (2012) |
|  | Interaction between number of living children and age (2012) |
| Health Status | Self-reported global health (2003 and 2012) |
|  | Has difficulty getting dressed (2003 and 2012) |
|  | Has difficulty walking across the room (2003 and 2012) |
|  | Has difficulty bathing in a tub or shower (2003 and 2012) |
|  | Has difficulty eating (2003 and 2012) |
|  | Has difficulty getting in and out of bed (2003 and 2012) |
|  | Has difficulty using the toilet (2003 and 2012) |
|  | Number of ADL limitations (2003 and 2012) |
|  | Has difficulty managing money (2003 and 2012) |
|  | Has difficulty taking medications (2003 and 2012) |
|  | Has difficulty shopping for groceries (2003 and 2012) |
|  | Has difficulty preparing a hot meal (2003 and 2012) |
|  | Number of IADL limitations (2003 and 2012) |
|  | Self-reported memory (2012) |
|  | Total number of ADL difficulties (2003 and 2012) |
|  | Binary indicator for any ADL difficulty (2003 and 2012) |
| Mental Health | Most of the past week, felt depressed (2003 and 2012) |
|  | Most of the past week, felt everything was an effort (2003 and 2012) |
|  | Most of the past week, felt that their sleep was restless (2003 and 2012) |
|  | Most of the past week, felt happy (2003 and 2012) |
|  | Most of the past week, felt lonely (2003 and 2012) |
|  | Most of the past week, felt that they enjoyed life (2003 and 2012) |
|  | Most of the past week, felt sad (2003 and 2012) |
|  | Most of the past week, felt tired (2003 and 2012) |
|  | Most of the past week, felt they had a lot of energy (2003 and 2012) |
|  | Number of depressive symptoms (CES-D) (2003 and 2012) |
|  | Binary indicator for 5+ CES-D depressive symptoms (2003 and 2012) |
|  | How much they agree with the statement that their life is close to ideal (2012) |
|  | How much they agree with the statement that life is excellent (2012) |
|  | How much they agree with the statement that they are satisfied with their life (2012) |
|  | How much they agree with the statement that they have achieved the things in life that are important to them (2012) |
|  | How much they agree with the statement that they would change almost nothing about their life (2012) |
|  | Positive emotion index (2003 and 2012) |
|  | Negative emotion index (2003 and 2012) |
|  | Emotional balance index (2003 and 2012) |
|  | Binary indicator for any negative emotion (2003 and 2012) |
| Diagnosis History | Diagnosed with hypertension (2003 and 2012) |
|  | Diagnosed with diabetes (2003 and 2012) |
|  | Diagnosed with respiratory illness (2003 and 2012) |
|  | Diagnosed with arthritis or rheumatism (2003 and 2012) |
|  | Told they had a heart attack (2003 and 2012) |
|  | Told they had a stroke (2003 and 2012) |
|  | Diagnosed with cancer (2003 and 2012) |
|  | Number of chronic illnesses (2003 and 2012) |
| Lifestyle & Social Interactions | Binned body mass index (BMI) (2003 and 2012) |
|  | Exercises 3 or more times per week (2003 and 2012) |
|  | Currently drinks alcohol (2003 and 2012) |
|  | Currently smokes tobacco (2003 and 2012) |
|  | Importance of religion (2003 and 2012) |
|  | Cares for sick or disabled adult (2012) |
|  | Cares for children under 12 (2012) |
|  | Volunteers for a non-profit (2012) |
|  | Attends training course, lecture, or class (2012) |
|  | Attends sports or social club (2012) |
|  | Reads books, magazines, or newspapers (2012) |
|  | Does crosswords, jigsaw puzzles, or number games (2012) |
|  | Plays tabletop games (e.g., cards, dominoes, chess) (2012) |
|  | Talks on the phone or uses the web (2012) |
|  | Maintains house, does repairs, gardens, etc. (2012) |
|  | Watches television (2012) |
|  | Sews, embroiders, knits, or makes crafts (2012) |
|  | Frequency of seeing friends and relatives (2012) |
|  | Frequency of participating in social activities (2012) |
|  | Participates in weekly religious services (2012) |
|  | Overall activity engagement score (2012) |
| Economic Status | Weekly work hours at main job (2003 and 2012) |
|  | Earnings from employment (2003 and 2012) |
|  | Spouse’s earnings from employment (2003 and 2012) |
|  | Household income (2003 and 2012) |
|  | Income from business (2003 and 2012) |
|  | Income from rent (2003 and 2012) |
|  | Income from financial assets (2003 and 2012) |
|  | Household capital income (2003 and 2012) |
|  | Income from pensions (2003 and 2012) |
|  | Spouse’s income from pensions (2003 and 2012) |
| Healthcare Utilization | Has had cholesterol test (2003 and 2012) |
|  | Has had tuberculosis test (2003 and 2012) |
|  | Has had diabetes test (2003 and 2012) |
|  | Has had high blood pressure test (2003 and 2012) |
|  | Has been hospitalized in the past year (2003 and 2012) |
|  | Has visited a doctor in the past year (2003 and 2012) |
|  | Has had an outpatient procedure (2003 and 2012) |
|  | Has visited a dentist in the past year (2003 and 2012) |
|  | Has health coverage with IMSS (2003 and 2012) |
|  | Has health coverage with ISSSTE (2003 and 2012) |
|  | Has health coverage with PEMEX, Defensa, or Marina (2003 and 2012) |
|  | Has private health insurance (2003 and 2012) |
|  | Has other health insurance (2003 and 2012) |
|  | Has Seguro Popular health coverage (2012) |
|  | Has any health insurance (2003 and 2012) |
|  | Vaccinated against flu (2012) |
|  | Vaccinated against pneumonia (2012) |
| Decision Making | Weight in family decisions (2003 and 2012) |
|  | Weight in personal decisions (2003 and 2012) |
|  | Employment status (2003 and 2012) |
| Life Changing Events | Change in marital status |
|  | Change in number of marriages |
|  | Change in number of living children |
|  | Change in U.S. migration status |
|  | Change in global health |
|  | Change in ADL and IADL difficulties |
|  | Change in CES-D depression symptoms |
|  | Change in diagnosed chronic illnesses |
|  | Change in BMI |
|  | Change in exercise frequency |
|  | Change in alcohol and tobacco use |
|  | Change in preventive test usage (cholesterol, diabetes, etc.) |
|  | Change in healthcare visits and insurance status |
|  | Change in decision-making roles |
|  | Change in employment status |
|  | Change in income |
|  | Change in perceived religious importance |

**Supplementary Table 1.** Feature grouping schema used for KNN-based prediction models. Features were grouped into nine categories—Demographics, Health Status, Mental Health, Diagnosis History, Lifestyle, Wealth, Healthcare Use, Decision Making, and Life Changing Events—based on their conceptual relevance and timing of collection (2003 and/or 2012). Descriptions are provided for interpretability and transparency in feature categorization.

| Feature names | Descriptions |
| --- | --- |
| iADL/ADL |  |
| imoney | Has difficulty managing money |
| eat | Has difficulty eating |
| dress | Has difficulty getting dressed |
| imeals | Has difficulty preparing a hot meal |
| ishop | Has difficulty shopping for groceries |
| bath | Has difficulty bathing themselves in a tub or shower |
| walk | Has difficulty walking from one side of the room to the other |
| toilet | Has difficulty using the toilet |
| bed | Has difficulty getting in and out of bed |
| imeds | Has difficulty taking medications |
| Emotional Well-being |  |
| happy | Most of the past week, felt happy |
| energetic | Most of the past week, felt they had a lot of energy |
| sad | Most of the past week, felt sad |
| tired | Most of the past week, felt tired |
| lonely | Most of the past week, felt lonely |
| depressed | Most of the past week, felt depressed |
| hard | Most of the past week, felt that everything was an effort |
| Engagement with Activities |  |
| reads | Uses time to read books, magazines, newspapers |
| sewing | Uses time to sew, embroider, knit, make crafts |
| games | Uses time to do crosswords, jigsaw puzzles, number games |
| table_games | Uses time to play tabletop games. E.g., cards, dominoes, chess |
| tv | Uses time to watch television |
| act_mant | Uses time to maintain a house, do repairs, garden, etc. |
| comms_tel_comp | Uses time to talk on the phone or send message/use the web on a computer |

**Supplementary Table 2.** Feature engineering summary for Activities of Daily Living (ADL), emotional well-being, and activity engagement.

| Model | Hyperparameters | Searching Range | Finalized Value |
| --- | --- | --- | --- |
| Lasso | regularization | [1e-4 to 1e2] (log-scale) | 0.1 |
| KNeighborsRegressor (grouped features) | n_neighbors | [2, 4, 8, 16, 32, 64] | 32 |
| Random Forrest | n_estimators | [50, 100, 200, 300, 400] | 100 |
|  | max_depth | [None, 10, 20, 30] | 10 |
|  | min_samples_split | [2, 5, 10] | 2 |
|  | min_samples_leaf | [1, 2, 4] | 1 |
| LightGBM Regressor | n_estimators | [500, 1500] (step=100) | 1400 |
|  | learning_rate | log-uniform [0.001, 0.01] | 0.0064 |
|  | num_leaves | [16, 128] (step=1) | 115 |
|  | max_depth | [15, 100] (step=1) | 53 |
|  | min_child_samples | [5, 30] (step=1) | 6 |
|  | subsample | [0.6, 1.0] | 0.861 |
|  | colsample_bytree | [0.6, 1.0] | 0.7453 |
|  | reg_alpha | [0, 1] | 0.1482 |
|  | reg_lambda | [0, 1] | 0.4076 |
|  | feature_fraction | [0.1, 1.0] | 0.372 |
|  | bagging_fraction | [0.1, 1.0] | 0.6825 |
|  | bagging_freq | [0, 10] (step=1) | 5 |
|  | min_data_in_leaf | [10, 200] (step=1) | 13 |
| XGBoost Regressor | n_estimators | [500, 1500] (step=100) | 1000 |
|  | learning_rate | log-uniform [0.05, 0.3679] | 0.0246 |
|  | max_depth | [3, 15] (step=1) | 13 |
|  | min_child_weight | [1, 10] (step=1) | 8 |
|  | subsample | [0.6, 1.0] | 0.5399 |
|  | colsample_bytree | [0.6, 1.0] | 0.5001 |
|  | reg_alpha | log-uniform [0.05, 1.0] | 0.2547 |
|  | reg_lambda | log-uniform [0.05, 1.0] | 0.3863 |
|  | gamma | [0, 5] | 3.6823 |
| Ridge | regularization | [1e-4 to 1e2] (log-scale) | 1 |

**Supplementary Table 3.** Hyperparameter search ranges and finalized values for each machine learning model evaluated in this study.
